# Identification of circRNA Expression Profile and Potential Systemic Immune Imbalance Modulation in Premature Rupture of Membranes

**DOI:** 10.1155/2024/6724914

**Published:** 2024-05-20

**Authors:** Dongni Huang, Yuxin Ran, Ruixin Chen, Jie He, Nanlin Yin, Hongbo Qi

**Affiliations:** ^1^Women and Children's Hospital of Chongqing Medical University (Chongqing Health Center for Women and Children), Chongqing, China; ^2^Chongqing Key Laboratory of Maternal and Fetal Medicine, Chongqing Medical University, Chongqing, China; ^3^Department of Gynecology and Obstetrics, West China Second Hospital, Sichuan University, Chengdu, China; ^4^Center for Reproductive Medicine, The First Affiliated Hospital of Chongqing Medical University, Chongqing, China

## Abstract

Premature rupture of membrane (PROM) refers to the rupture of membranes before the onset of labor which increases the risk of perinatal morbidity and mortality. Recently, circular RNAs (circRNAs) have emerged as promising regulators of diverse diseases. However, the circRNA expression profiles and potential circRNA–miRNA–mRNA regulatory mechanisms in PROM remain enigmatic. In this study, we displayed the expression profiles of circRNAs and mRNAs in plasma and fetal membranes of PROM and normal control (NC) groups based on circRNA microarray, the Gene Expression Omnibus database, and NCBI's Sequence Read Archive. A total of 1,459 differentially expressed circRNAs (DECs) in PROM were identified, with 406 upregulated and 1,053 downregulated. Then, we constructed the circRNA–miRNA–mRNA network in PROM, encompassing 22 circRNA–miRNA pairs and 128 miRNA–mRNA pairs. Based on the analysis of gene ontology (GO) and the Kyoto Encyclopedia of Genes and Genomes (KEGG) pathway and gene set enrichment analysis (GSEA), DECs were implicated in immune-related pathways, with certain alterations persisting even postpartum. Notably, 11 host genes shared by DECs of fetal membrane tissue and prenatal plasma in PROM were significantly implicated in inflammatory processes and extracellular matrix regulation. Our results suggest that structurally stable circRNAs may predispose to PROM by mediating systemic immune imbalances, including peripheral leukocyte disorganization, local immune imbalance at the maternal–fetal interface, and local collagen disruption. This is the first time to decipher a landscape on circRNAs of PROM, reveals the pathogenic cause of PROM from the perspective of circRNA, and opens up a new direction for the diagnosis and treatment of PROM.

## 1. Introduction

Premature rupture of membranes (PROM) is defined as the rupture of the fetal membranes before the onset of labor which complicates ∼8% of pregnancies worldwide [1, 2]. PROM before 37 weeks of gestation occurs in around 3% of all pregnancies, was significantly associated with more severe perinatal complications such as premature delivery, chorioamnionitis, and placental abruption, and is the main cause of maternal perinatal death [3, 4]. It is generally accepted that PROM is a multifactorial disease with multiple causes (e.g., infection and endocrine disruption) [5, 6], and the biological changes of membranes are the core pathological basis of PROM, including matrix degradation, cell senescence, apoptosis, autophagy, and epithelial–mesenchymal transition [7, 8]. However, as the most common perinatal disease, research on PROM often focused on the prediction of severe maternal–fetal outcomes via common biomarkers [9, 10], and the core etiology and key molecular mechanism of PROM remain unclear. Notably, noncoding RNAs (ncRNAs) have brought new light to PROM research, represented by miRNAs [11] and long noncoding RNAs (lncRNAs) [12]. Therefore, we speculated that the novel and more powerful ncRNA molecule, circRNA, could better decipher the enigma of PROM.

Circular RNA (circRNA) is a newly identified special class of ncRNA molecules with a covalently closed loop structure and great biofunctions [13, 14]. With the features of great abundance, high stability, tissue- and developmental-stage specificity, and wide distribution in the body, circRNA could greatly reflect the morbid status [15, 16]. The molecular mechanisms and future value of circRNA in participating in and regulating biological and pathological processes are also becoming increasingly apparent [17]. The ceRNA hypothesis proposes that circRNA with miRNA response elements (MREs) can act as an endogenous miRNA sponge to bind to miRNA and regulate its function, thereby regulating the expression level of downstream proteins [18]. Strikingly, recent studies have proved that circRNA, as a competing endogenous RNA (ceRNA), has a powerful regulatory effect in the pathological processes of many diseases [19–22], especially immune imbalances and extracellular matrix [23–25]. Besides, circRNA could improve the prediction efficiency of traditional prediction models composed of classical indexes (neutrophil to lymphocyte (NLR), platelet to lymphocyte (PLR), and lymphocyte to monocyte (LMR) ratios) [26]. It provides a good theoretical basis for early prediction and precise intervention of many diseases.

Although circRNA has not been adequately studied in the perinatal field, its extraordinary biological activities and clinical significance in gestational diseases have already been shown [27–29]. For instance, circPUM1 could impair recurrent spontaneous abortion occurrence and protect against inflammation via the miR-30a-5p/JUNB axis [30]. The circ_0001861/miR-296-5p/FOXP1 axis plays a regulating role in trophoblast cell proliferation, migration, invasion, and EMT in pre-eclampsia [31]. circ_0001578 promotes gestational diabetes mellitus by inducing placental inflammation [32]. Meanwhile, our previous study also revealed for the first time that the disturbance of circRNA expression in the maternal–fetal system may induce preterm labor by mediating immune imbalance [33]. These studies reveal that circRNA often mediates pregnancy complications and immune imbalance in maternal circulation and can also cause diseases locally at the maternal–fetal interface by affecting cellular function. Despite this increase in interest, the expression and biological function of circRNAs in PROM have been reported rarely. Therefore, this study aims to depict the expression profiles of circRNAs in PROM via the RNA-seq data and microarray data. Our work will discover the circRNA disorders in PROM for the first time, enrich the mechanistic theory of PROM, and may provide a new strategy for its diagnosis and treatment.

## 2. Materials and Methods

### 2.1. Sample Collection and Preparation

In this case–control study of singleton, pregnant women were admitted to the First Affiliated Hospital of Chongqing Medical University in China between April 2019 and January 2020. The normal control (NC) group was composed of healthy pregnancies with intact fetal membranes and not afflicted by gestational diseases. The inclusion criteria for the PROM group were as follows: (1) patients with rupture of membranes before the onset of labor; (2) without PE, GDM, and other severe gestational complications. The characteristics of all participants are summarized in Table 1. All cases identified were matched 1 : 1 to randomly selected controls. When they were admitted to the hospital for delivery, ∼5 ml of blood was collected into EDTA-treated tubes. Then, the blood samples were centrifuged at 3,000 rpm for 15 min at 4°C to retrieve plasma, which was subsequently stored at −80°C until assaying. In total, four paired peripheral blood plasma samples from the NC and PROM groups were collected.

All participants provided written informed consent. The protocols complied with the Helsinki Declaration (World Medical Association Declaration of Helsinki).

### 2.2. circRNA Microarray

The total RNA of plasma was extracted using TRIzol Reagent (Invitrogen, Gaithersburg, MD, USA) for microarray analysis as previously described [34]. Briefly, RNA was digested with RNase R, amplificated, labeled, purified, and quantified according to the manufacturer's protocol. Then, chip hybridization was performed using Agilent Human circRNA Array (V2.0), which contains probes interrogating about 170,340 human circRNAs in CapitalBio company (Beijing, China). After being washed, the arrays were scanned on the Agilent Microarray scanner (G2565C). Agilent Feature Extraction (V10.7) software and Agilent GeneSpring software were used to analyze the data. All tests were completed by January 2020.

### 2.3. External Datasets Collection

The mRNA expression data of four PROM and four NC blood samples (GSE212859) and the miRNA expression data of three PROM and three NC blood samples (GSE73685) were obtained from the Gene Expression Omnibus (GEO) database (https://www.ncbi.nlm.nih.gov/geo/). The whole-transcriptome sequencing raw data of four PROM and four NC fetal membrane samples (SRP139931) were downloaded from NCBI Sequence Read Archive (SRA) (https://www.ncbi.nlm.nih.gov/sra/) databases. The circRNAs were identified and quantified by the CIRIquant software with default settings. In this step, the genome file and the annotation file were the UCSC human reference genome (hg38) and gencode.v42.annotation.gtf.

### 2.4. Differential Analysis

The microarray data were preprocessed by log2-transformation and quantile normalization. Subsequently, the differential analysis was performed by the R (version 3.6.2) package “Limma.” For RNA-seq count data, the differential analysis was performed by the R package “DEseq2.” The selection threshold was set to fold change (FC) > 2.0 and *P*-value < 0.05.

### 2.5. Support Vector Machines (SVMs)

SVM, a supervised machine learning algorithm, was performed using the R packages “e1071” and “caret” to initially screen genes that contribute significantly to the differences between groups. The “importance” function was used to rank the variables by importance. The *P*-value < 0.05 was considered significant.

### 2.6. Construction of circRNA–miRNA–mRNA Networks

The target miRNAs of key circRNAs were predicted by circBank which integrates the result data from miRanda and TargetScan (http://www.targetscan.org/) based on the miRNA binding site. Then, the downstream mRNAs of miRNAs were predicted by miRDB (http://www.mirdb.org/) and TargetScan.

The circRNA–miRNA–mRNA networks were built using Cytoscape (version 3.6.1).

### 2.7. Functional Enrichment Analysis

The GO (gene ontology) and the KEGG (Kyoto Encyclopedia of Gene and Genomes) pathway enrichment analyses were conducted using the R package “Clusterprofiler.” GO terms or KEGG pathways with *P*-value < 0.05 were considered significant. Then, the results were visualized by the “ggplot2” package of R software.

### 2.8. Gene Set Enrichment Analysis (GSEA)

This analysis was completed by the GSEA software (version 4.0.3) obtained from the Broad Institute (http://www.broadinstitute.org/gsea). The gene sets of biological processes were downloaded from the Molecular Signatures Database (http://software.broadinstitute.org/gsea/msigdb). The selection threshold was set to normalized enrichment score (NES) > 0 and *P*-value < 0.05.

### 2.9. Statistical Analysis

Statistical analysis was performed using the SPSS (version 25.0, Chicago, IL, United States), GraphPadPrism (version 8.0, San Diego, CA, United States), and R (version 3.6.2) software.

The mean and standard deviation (mean ± SD) of all data were calculated. Student's *t*-test was used for variable data analysis, and Fisher's exact tests were used for statistical analysis of categorical variables. The Pearson correlation analysis was performed by the R software (version 3.6.2).

## 3. Results

### 3.1. The circRNAs in Prenatal Plasma of PROM and NC Pregnancies

**Figure 1:**
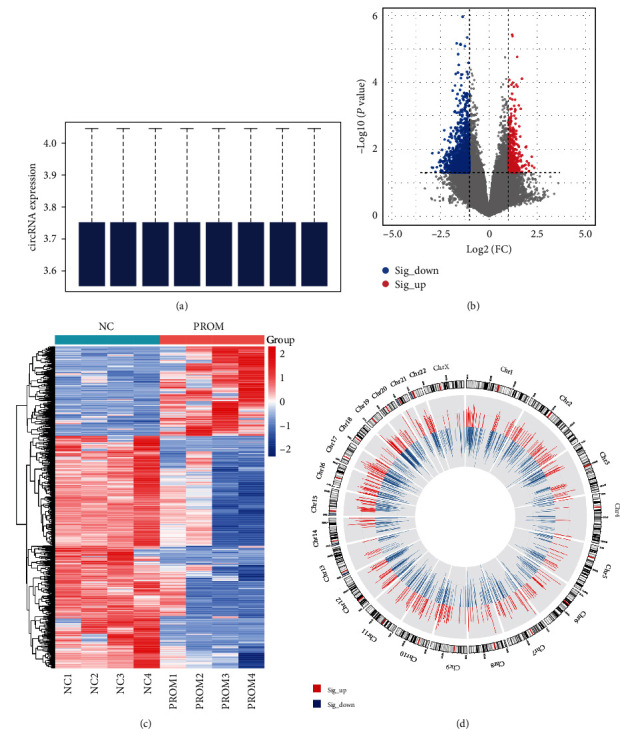
The differentially expressed circRNAs between the PROM and NC groups before delivery: (a) the normalized circRNA expression level (log2-transformed signal intensity) in each sample; (b) DEcircRNAs with FC > 2.0 and *P*-value < 0.05; (c) the expression profiles of DEcircRNAs in PROM and NC groups before delivery; (d) chromosomal distribution pattern of DEcircRNAs.

The circRNA microarray analysis was performed to identify DEcircRNAs in the peripheral plasma of PROM and NC pregnancies. After data clean and upper quartile normalization, 99,287 circRNAs were screened out (Figure 1(a)). In total, 1,459 circRNAs were significantly differentially expressed in PROM compared to the NC group, among which 406 circRNAs were upregulated and 1,053 circRNAs were downregulated (Figures 1(b) and 1(c)). The genomic position from which these circRNAs derived is shown in Figure 1(d), and no evident aggregation phenomenon was observed.

### 3.2. The Key circRNAs and Their Regulatory Network in Prenatal Plasma of PROM

**Figure 2:**
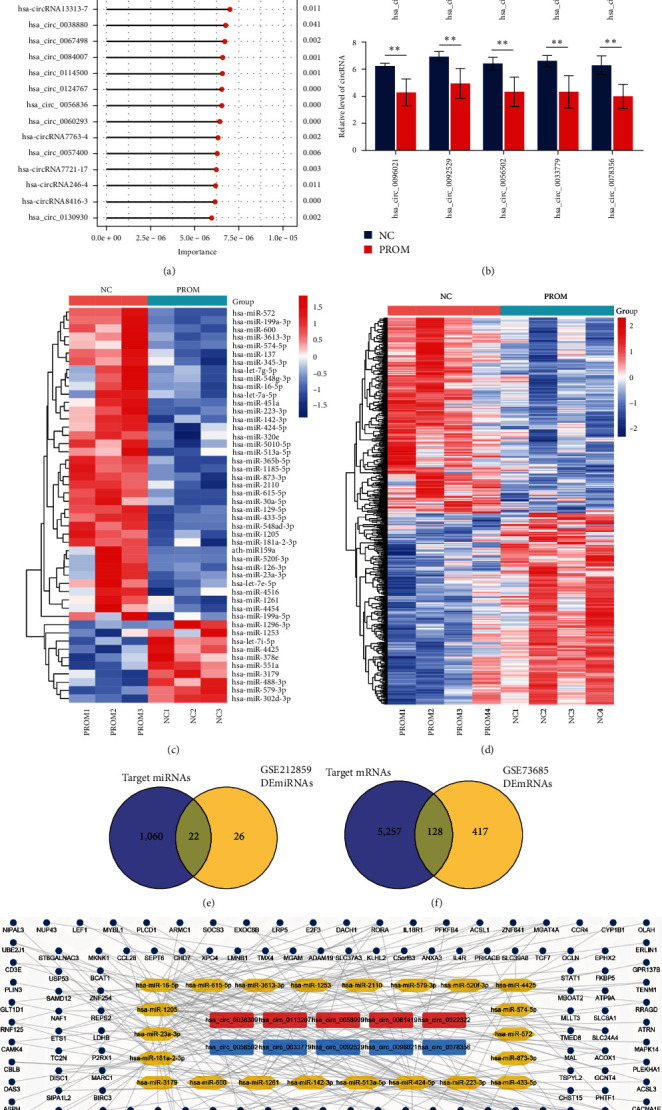
The top DEcircRNAs and their regulated network: (a) the top 20 circRNAs which are selected by the SVM algorithm (*P*-value < 0.05); (b) the expression level of the top circRNAs in the PROM and NC groups ( ^*∗*^*P*-value < 0.05;  ^*∗∗*^*P*-value < 0.01;  ^*∗∗∗*^*P*-value < 0.001); (c) the expression profiles of DEmiRNAs between the PROM and NC groups (FC > 2 and *P*-value < 0.05); (d) the expression profiles of DEmRNAs between the PROM and NC groups (FC > 1.5 and *P*-value < 0.05); (e) the intersection of target miRNAs and DEmiRNAs; (f) the intersection of target mRNAs and DEmRNAs; (g) the ceRNA (circRNA–miRNA–mRNA) regulatory network of top circRNAs in PROM (red squares: upregulated circRNAs; blue squares: downregulated circRNAs; yellow hexagons: miRNAs; dark blue circles: mRNAs).

Using the SVM algorithm, 140 candidate circRNAs that contributed significantly to the classification of the NC and PROM groups were screened from the DEcircRNAs (Figure 2(a)). Considering the fold change and importance value of these circRNAs, we selected the up- and down-regulated top 5 circRNAs, which may have a greater impact on the pathological process of PROM (Figure 2(b) and Table 2). A total of 48 miRNAs and 545 mRNAs that significantly dysregulated in the circulation of PROM patients were identified from two independent datasets (GSE212859 and GSE73685), respectively (Figures 2(c) and 2(d)). Based on the miRNA binding site, 1,082-targeted miRNAs of key circRNAs were predicted. Then, 22 miRNAs bound by top circRNAs in PROM were obtained through the intersection of the targeted and dysregulated miRNAs (Figure 2(e)). Similarly, the 128 downstream mRNAs regulated by top circRNAs via 22 miRNAs were identified (Figure 2(f)). Thus, the regulatory network of the key circRNAs consisting of 22 circRNA–miRNA pairs and 128 miRNA–mRNA pairs was constructed in PROM.

### 3.3. The Biofunctions of Key circRNAs in Prenatal Plasma of PROM

**Figure 3:**
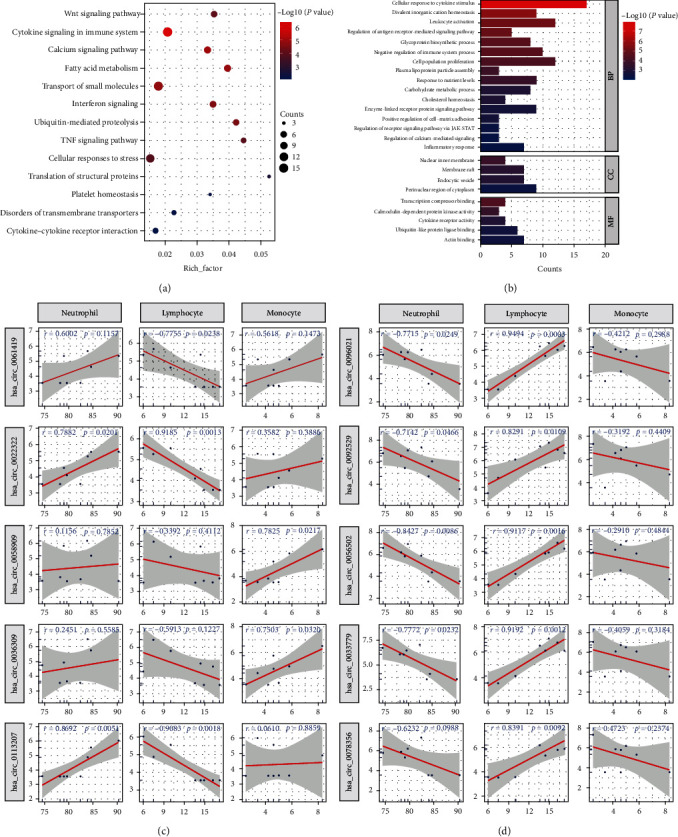
The top circRNAs-related biological processes: (a) the enriched KEGG terms of genes which are regulated by the top circRNAs; (b) the enriched GO terms of genes which are regulated by the top circRNAs. The linear regression analysis for the immune cells and upregulated (c) and downregulated (d) circRNAs.

To investigate the biological role of key circRNAs, annotation and enrichment analyses were performed for the downstream genes regulated by these circRNAs via the ceRNA mechanism. These genes are enriched in immune-inflammatory and energy metabolism pathways represented by “cytokine signaling in immune system,” “cytokine–cytokine receptor interaction,” “fatty acid metabolism,” etc. (Figure 3(a)). Consistent with this, they are also involved in GO biological processes such as “leukocyte activation,” “cellular response to cytokine stimulus,” “carbohydrate metabolic process,” etc. In addition, the terms related to cell adhesion as well as extracellular matrix are also noteworthy (Figure 3(b)).

The leukocyte levels in the prenatal peripheral blood of PROM patients appeared to be disordered, especially lymphocytes (Table 3). Through linear regression analysis, we found that the levels of key circRNAs were significantly correlated with the percentage of neutrophils, lymphocytes, and monocytes (|*r*| > 0.7 and *P*-value < 0.05) (Figures 3(c) and 3(d)).

### 3.4. The circRNAs in Postnatal Plasma of PROM and NC Pregnancies

**Figure 4:**
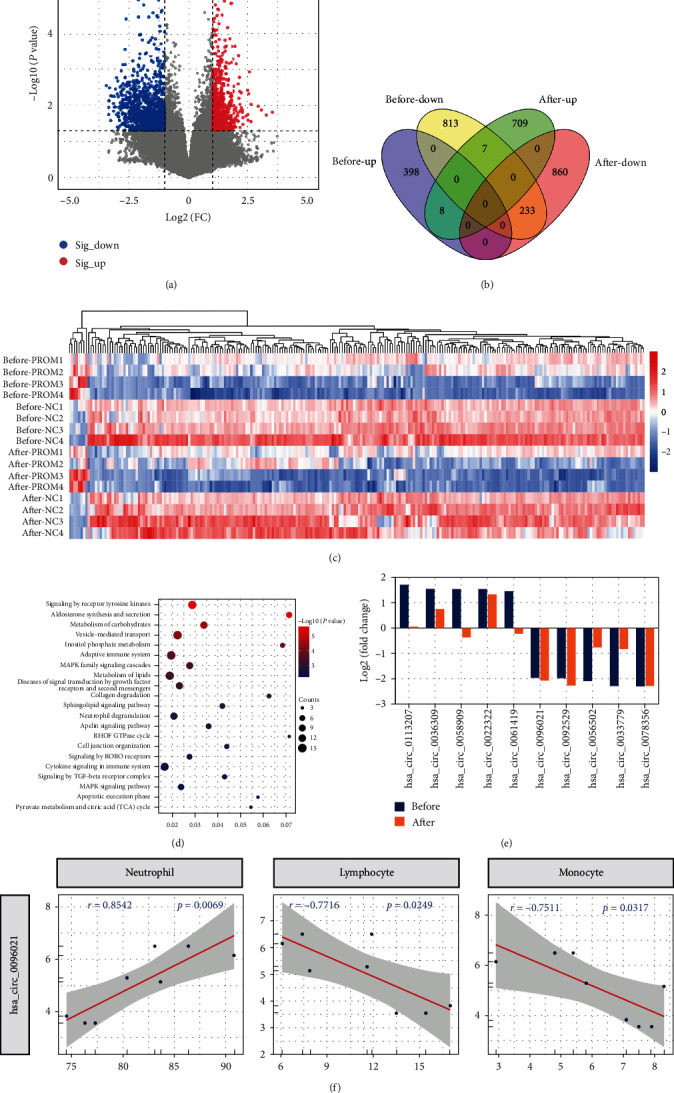
The differentially expressed circRNAs between the PROM and NC groups after delivery: (a) DEcircRNAs with FC > 2.0 and *P*-value < 0.05; (b) the intersection of DEcircRNAs of prenatal and postnatal samples; (c) the expression profiles of intersection DEcircRNAs; (d) the intersection circRNAs-related biological processes; (e) the log2FC of the top circRNAs in prenatal and postnatal samples; (f) the linear regression analysis for the immune cells and has_circ-0096021.

Interestingly, even after delivery (26.25 ± 14.95 hr), the plasma circRNA expression profiles of PROM and NC groups were still different. There were 724 up- and 1,093 down-regulated circRNAs in PROM (Figure 4(a)). Of these, 233 downregulated and 8 upregulated circRNAs were present with the same change pattern in prenatal plasma (Figures 4(b) and 4(c)). All these 241 circRNAs were related to “metabolism of carbohydrates,” “adaptive immune system,” “collagen degradation,” etc. (Figure 4(d)). This suggests that the effect of circRNAs on immune-inflammatory, energy metabolism, and extracellular matrix in PROM may persist from the prenatal to the postnatal period.

Of the 10 key circRNAs selected prenatally, three remained significantly dysregulated in PROM postnatally: hsa_circ_0096021, hsa_circ_0092529, and hsa_circ_0078356 (Figure 4(e)). Notably, hsa_circ_0096021 remained strongly linearly associated with postnatal levels of peripheral blood neutrophils, lymphocytes, and monocytes (|*r*| > 0.7 and *P*-value < 0.05) (Figure 4(f) and Table 4).

### 3.5. The Disordered circRNAs in the Circulation and Fetal Membrane of PROM

**Figure 5:**
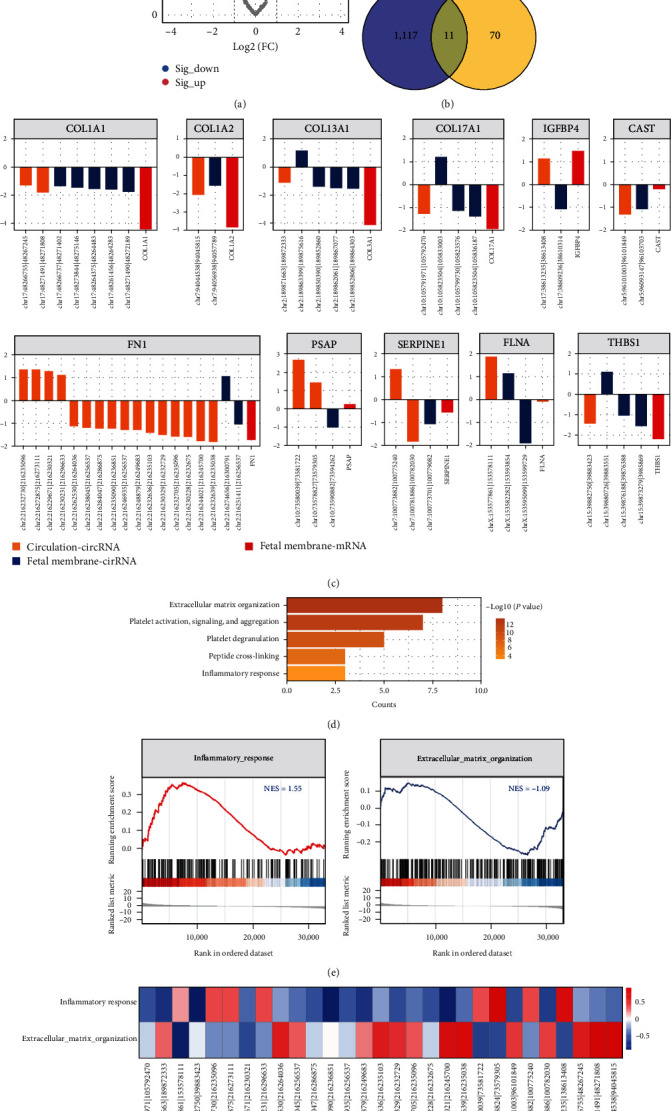
The differentially expressed circRNAs in the fetal membrane between the PROM and NC groups: (a) DEcircRNAs with FC > 2.0 and *P*-value < 0.05; (b) the intersection of DEcircRNAs of circulation and fetal membrane; (c) the log2FC of the intersection DEcircRNA and mRNAs derived from their host genes; (d) the intersection circRNAs-related biological processes; (e) the significantly dysregulated biological processes in the fetal membrane of PROM; (f) the correlations between the intersection circRNAs and the significantly dysregulated biological processes in the fetal membrane of PROM (the colors indicate the correlation coefficients).

In the fetal membrane of PROM and NC pregnancies, we identified 119 DEcircRNAs, 68 and 51 of which were up- and down-regulated in PROM, respectively (Figure 5(a)). Furthermore, we found that 11 host genes were shared by DEcircRNAs of fetal membrane tissue and prenatal plasma in PROM (Figure 5(b)). The circRNAs and mRNAs derived from these 11 host genes might be the mediator of communication between the circulation and fetal membrane in PROM. Interestingly, about half of the host gene encodes fibronectin and collagen (Figure 5(c)).

The circRNAs from these host genes were involved in the processes of inflammatory and extracellular matrix, while the “INFLAMMATORY_RESPONSE” was activated and the “EXTRACELLULAR_MATRIX_ORGANIZATION” was inhibited in PROM (Figures 5(d) and 5(e)). Finally, the linear regression analysis confirmed that these mediator circRNAs were inextricably linked to the essential immune inflammation and extracellular matrix processes in the circulation and fetal membrane of PROM (Figure 5(f)).

## 4. Discussion

To our knowledge, systemic immune imbalances may be responsible for the development of PROM, but its pathogenesis remains largely unclear [35]. It is necessary to research the pathogenesis of PROM and search for its biomarkers. Recent research on circRNA in gestational diseases has achieved considerable development and breakthroughs [36–38]. Nevertheless, the circRNAs expression and functions in PROM remain completely unknown. To address this knowledge gap, we investigated the circRNAs expression profile in plasma from PROM and NC pregnant women and identified 1,459 DEcircRNAs. Then, the regulatory network of the key circRNAs consisting of 22 circRNA–miRNA pairs and 128 miRNA–mRNA pairs was constructed. These DEcircRNAs mainly mediated peripheral leukocyte disorder expression which has been elucidated as vital parts of PROM pathogenesis mechanisms. Interestingly, there are no studies currently available about the dysregulation and functions of these circRNAs in diseases, indicating that they may have unique associations with PROM. Noteworthy, the effect of circRNAs on immune-inflammatory, energy metabolism, and extracellular matrix in PROM may persist from the prenatal to the postnatal period.

Due to the lack of accurate and early prediction of the outbreak window, we can hardly intervene early in the occurrence of premature rupture of membranes [39, 40], and only empiric treatment after the occurrence of the disease can maximize the maternal and fetal outcomes; as a classical obstetric practice, there have been no significant changes for the last many years [4]. Therefore, we are committed to finding the key to initiate premature rupture of membranes. This study suggests that maternal systemic immune disorder breaks out prematurely before delivery, and the maternal–fetal interface receives false signals, which leads to local immune imbalance and premature rupture of fetal membranes. Finding the sentinel point of the outbreak of maternal systemic immune disorders has become a top priority.

As a starting point for systemic immune disorders, we focus on peripheral white blood cells. Neutrophils are often exclusively considered as a first-line innate immune defense, able to rapidly kill or trap pathogens, and cause in case of overactivation tissue damage [41]. Increased maternal neutrophil may mediate chronic low-grade inflammation in PROM [9, 42], gestational diabetes mellitus [43, 44], and pre-eclampsia [45]. Neutrophil infiltration is also a key cause of fetal membrane inflammation and tissue destruction at the maternal–fetal interface [46]. On the other hand, the functional significance of lymphocytes in pregnancy was affirmed by a huge number of studies. Since embryo implantation, the lymphocytes at the maternal–fetal interface begin to work for the maintenance of pregnancy [47]. The abnormal distribution of lymphocytes may directly lead to the occurrence of maternal–fetal interface infection, or lead to other complications of pregnancy such as recurrent abortion [48, 49] and pre-eclampsia [50] after a long period of immune microenvironment changes. This abnormal distribution in pregnancy complications can also be observed in the peripheral blood [51]. During pregnancy, the communication between the mother and the child may be reflected in the status of peripheral blood mononuclear cells [52]. Pregnancy comes with increased number, phagocytic activity, and ROS production capacity of monocytes [53]. Once the balance maintained by monocytes during pregnancy is broken [54], it may induce the release of proinflammatory factors, which may induce premature rupture of membranes [55], premature delivery [56], and other common complications of pregnancy. Our results suggested circRNA may directly lead to immune imbalance by mediating abnormal secretion of cytokines and may also lead to the imbalance of peripheral leukocyte distribution through the synergistic effect of cytokines, resulting in PROM finally. This provides a novel explanation for the regulatory effect of circRNAs on peripheral blood leukocytes and further expands the etiological evidence for immune imbalance in PROM.

Considering the unique stability of circRNA [57], this study sequentially concerns the continuous regulation of circRNA in the mother after delivery. The results suggest that among the 10 key circRNAs selected prenatally, 3 are still significantly dysregulated in PROM after birth: hsa_circ_0096021, hsa_circ_0092529, and hsa_circ_0078356. Among them, hsa_circ_0096021 maintains a strong linear correlation with the level of peripheral blood leukocytes after birth, which may be due to the need for maternal immune maintenance and tissue repair after delivery [58]. Meanwhile, circRNA also participates in maternal metabolic function, which plays an important role in maternal recovery after delivery [59]. Whether these circRNAs exist in the body of pregnant women with premature rupture of membranes for a long time, regulate other functions of the human body, or even lead to more diseases is not clear.

Rupture of fetal membranes is the moment of the outbreak of local inflammation of fetal membranes [60] and tissue collagen recombination degradation [61] and finally lead to structural destruction. Therefore, we also analyzed the function of local circRNA in the fetal membrane. We found that 11 host genes were shared by DEcircRNA in fetal membrane tissue and prenatal plasma in PROM, which suggested that these circRNAs may act as communication mediators, transmitting signals of peripheral immune imbalance to the local fetal membrane and opening local immune disorder in fetal membrane tissue. Moreover, about half of the host genes encode fibronectin and collagen. It can be explained that these circRNAs act as communication sentinels, which not only open the disorder of peripheral blood leukocytes but also accurately transmit the signal to the local fetal membrane, mediate the degradation and reorganization of local tissue collagen, and finally lead to the collapse of fetal membrane structure. At this point, premature rupture of membranes occurred.

This study still has some limitations. First, we analyzed the circRNAs in PROM via bioinformatics methods, but given the complexity of biological activities in vivo, the actual specific functions of circRNAs should be further in-depth explored and validated. Second, studies with a larger sample size are needed to verify our results better. Thereby, more systematic studies would be performed to gradually reveal the complex roles of circRNAs in PROM in the future.

In conclusion, this study revealed the DECs in PROM for the first time and suggested that these circRNAs may modulate the abnormal distribution of peripheral blood leukocytes via circRNA–miRNA–mRNA mechanisms, then resulting in PROM. Our study provides a novel insight into the pathogenesis for PROM from circRNA's view, and further studies are warranted to investigate the specific regulatory mechanisms of circRNAs in PROM.

## Figures and Tables

**Table 1:** The clinical characteristics of participants.

Characteristics	PROM (*n* = 4)	NC (*n* = 4)	*P* value
Age (years)	27.25 ± 0.50	32.25 ± 4.19	0.0557
BMI (kg/m^2^)	26.50 ± 2.43	30.85 ± 4.29	0.1277
Sampling 1 (gestational weeks)	33.93 ± 3.70	35.35 ± 4.75	0.6527
Delivery (gestational weeks)	34.28 ± 3.58	35.55 ± 4.74	0.6827
Sampling 2 (hr after delivery)	22.25 ± 16.86	30.25 ± 13.96	0.4924
History of PROM	0	0	—
Delivery modes	4	4	0.4857
Vaginal	3	1	—
Cesarean	1	3	—

**Table 2:** The details of the top 5 upregulated and downregulated circRNAs in the PROM group.

Circbase_ID	Chr	Start	End	Strand	Length	Deepbase_ID	Host_Gene	SVM		DE	
								Importance	*P* value	Log2FC	*P*_value
hsa_circ_0113207	chr1	36,335,336	36,354,211	+	13,895	−	AGO1	5.30E-06	0.0015	1.6995	0.0001
hsa_circ_0036309	chr15	75,140,940	75,165,670	−	808	hsa-circRNA10825-2	SCAMP2	4.17E-06	0.0030	1.5445	0.0005
hsa_circ_0058909	chr2	240,098,108	240,274,613	−	709	hsa-circRNA12935-3	HDAC4	7.05E-06	0.0016	1.5331	0.0230
hsa_circ_0022322	chr11	61,118,462	61,124,843	−	889	hsa-circRNA9507-4	CYB561A3	3.93E-06	0.0052	1.5299	0.0168
hsa_circ_0061419	chr21	33,057,361	33,064,762	−	975	hsa-circRNA13167-22	SCAF4	2.23E-06	0.0229	1.4483	0.0207
hsa_circ_0096021	chr11	61,115,401	61,115,528	+	127	−	DAK	2.80E-06	0.0185	−1.9662	0.0034
hsa_circ_0092529	chr10	105,330,628	105,344,982	+	1,254	−	NEURL1	4.33E-06	0.0301	−1.9903	0.0062
hsa_circ_0056502	chr2	132,442,469	132,443,784	+	1,315	hsa-circRNA4637-5	−	2.95E-06	0.0396	−2.0929	0.0055
hsa_circ_0033779	chr14	106,331,860	106,348,701	−	16,841	hsa-circRNA10608-230	−	2.01E-06	0.0323	−2.2842	0.0051
hsa_circ_0078356	chr6	155,054,928	155,155,194	+	4,587	hsa-circRNA6940-1	SCAF8	2.31E-06	0.0343	−2.2959	0.0027

**Table 3:** Routine blood examination before delivery.

Indicators	PROM (*n* = 4)	NC (*n* = 4)	*P* value
WBC (10^9^/L)	15.26 ± 7.05	11.56 ± 3.39	0.3804
RBC (10^12^/L)	3.95 ± 0.35	4.31 ± 0.37	0.2039
Hb (g/L)	128.75 ± 11.09	126.50 ± 11.82	0.7906
PLT (10^9^/L)	196.25 ± 38.81	170.25 ± 57.05	0.4796
NEUT%	84.45 ± 4.63	78.68 ± 3.29	0.0880
NEUT# (10^9^/L)	13.09 ± 6.62	9.16 ± 3.00	0.3207
LYM%	9.48 ± 3.67	15.48 ± 1.56	0.0239
LYM# (10^9^/L)	1.27 ± 0.17	1.76 ± 0.39	0.0619
MONO%	5.53 ± 2.09	4.08 ± 1.10	0.2660
MONO# (10^9^/L)	0.83 ± 0.51	0.46 ± 0.15	0.2137
EO%	0.38 ± 0.32	1.55 ± 2.02	0.2944
BASO%	0.18 ± 0.05	0.23 ± 0.26	0.7216

WBC, white blood cells; RBC, red blood cells; Hb, hemoglobin; PLT, platelets; NEUT%, neutrophil percentage; NEUT#, neutrophil counts; LYM%, lymphocyte percentage; LYM#, lymphocyte counts; MONO%, monocyte percentage; MONO#, monocyte counts; EO%, eosinophil percentage; BASO%, basophil percentage.

**Table 4:** Routine blood examination after delivery.

Indicators	PROM (*n* = 4)	NC (*n* = 4)	*P* value
WBC (10^9^/L)	13.97 ± 4.61	14.12 ± 3.09	0.9580
RBC (10^12^/L)	3.78 ± 0.49	4.02 ± 0.55	0.5375
Hb (g/L)	123.00 ± 9.76	120.50 ± 14.93	0.7887
PLT (10^9^/L)	190.75 ± 50.59	181.75 ± 52.77	0.8137
NEUT%	77.95 ± 4.00	85.20 ± 4.52	0.0533
NEUT# (10^9^/L)	11.01 ± 4.23	12.11 ± 3.12	0.6901
LYM%	13.45 ± 3.97	9.25 ± 2.94	0.1397
LYM# (10^9^/L)	1.77 ± 0.38	1.25 ± 0.22	0.0554
MONO%	7.70 ± 0.52	4.73 ± 1.28	0.0051
MONO# (10^9^/L)	1.09 ± 0.43	0.66 ± 0.21	0.1187
EO%	0.68 ± 0.67	0.70 ± 0.96	0.9671
BASO%	0.23 ± 0.19	0.13 ± 0.13	0.4128

WBC, white blood cells; RBC, red blood cells; Hb, hemoglobin; PLT, platelets; NEUT%, neutrophil percentage; NEUT#, neutrophil counts; LYM%, lymphocyte percentage; LYM#, lymphocyte counts; MONO%, monocyte percentage; MONO#, monocyte counts; EO%, eosinophil percentage; BASO%, basophil percentage.

## Data Availability

All data generated for this study are included in the article, and further inquiries can be directed to the corresponding author.
